# Physiological Reflections on the Development of the Human Teeth and the Reciprocal Effects of the Roots of the Milk Teeth

**Published:** 1857-07

**Authors:** Moritz Franco

**Affiliations:** Sub-Surgeon of the Austrian Imperial Army, at Mainz.


### ARTICLE II.

*Physiological Reflections on the Development of the Human Teeth and the reciprocal effects of the Roots of the Milk Teeth.* By MORITZ FRANCO, Sub-Surgeon of the Austrian Imperial Army, at Mainz. Translated from the German, from DER ZAHNARZT, for Am. Jour. Dent. Science.

THE object of the present essay is to point out the application of the great law of motion and change of matter, (as established by the latest and best works on physiology,) to the phenomena of the short life of the so-called milk teeth.

The origin and decay of all organic structures depends upon the reciprocal action of extremely minute particles, which perpetually unite and separate. This is termed the change of matter.

"An expression like this properly excites an emotion of awe, for as trade is the soul of intercourse, so is the permanent circulation of matter the soul of the world. Because the quantity of matter is liable to neither increase nor diminution, its qualities are determined from all eternity. The excrements of man nourish the plant; the plant metamorphoses the air into solid compounds and feeds the animal; beasts of prey devour vegetable feeders, to become themselves subject to death and to spread a new budding life throughout the world of plants." (J. Moleschott.)

"Nothing can be lost in a system, where every thing by turns attracts and is attracted. The quantity of existing matter is always the same." (G. Forster.)

These undeniable propositions prove, that the origin, the growth, the highest degree of development and finally the retrogressive metamorphosis, especially of the forms of the animal world, are inseparable from life. This fluctuation of development and decay is called by J. Moleschott, the motion of the elements, and he thus expresses himself touching the immortality of matter: "Motion of the elements, combination and decomposition, ingestion and excretion are the sum of all action upon earth. Activity is called life, if a body preserves its form and general composition in spite of perpetual changes of the most minute elementary parts which compose it. For this reason we talk about change of matter with regard to living beings. The lifeless body, the rock, weathers and changes its form. Change of material and weathering are discriminating differences between living and lifeless formations." In these words of fresh thought and truth may be found the explanation of all physiological activity.

After this introduction, which shows, that no process in such formations as we are capable of examining can find any reasonable explanation except on grounds like these, I come to the motive which induced me to write these remarks on the absorption of the roots of the milk teeth.

There exist on this subject a great many opinions and all of the most contradictory character. Many refused to accept as a foundation that earliest exposition of this change, that the blood-vessels of the milk teeth being compressed by the growth of the teeth of replacement become more or less impermeable to the blood, that mother liquid, out of which every thing is formed in the animal organism, although such views might have led to better results by careful investigation.

This first of all facts enumerates even men of prominence amongst its opponents. They abandoned rational opinions and long established facts, thereby coming into conflict with science, and hunted phantoms in the regions of fancy, which they either never could reach, or if they succeeded by high flight of thought, the toilsomely acquired opinion changed to nothing. Strangely do those parties declare the disappearance of the roots of the milk teeth, to say nothing about the excavation of the crowns, an event nowhere repeated in a living body; although similar occurrences are very frequent, as I shall show further on.

Philosophy shows, that development and decay are inseparable from life. If I apply this proposition in the narrowest sense to the formation of the animal body, I see, not to wander too far in generalities, how the first beginning of a man exists in an extremely small, hardly perceptible, bleb or egg. With what vigor the imbibition of materials and their union in new compounds are carried on, is evident from the fact that within a space of forty weeks from conception the fœtus reaches the full size of a child ready for birth. This appropriation of matter continues to the fortieth year of man's life, though the greatest activity of nutrition and growth belongs to the first half of this period.

Once having attained this its acme of development, metamorphosis undergoes a revolution. Hitherto absorption and assimilation have been predominant and have produced a great increase of bodily substance; but now diminished ingestion and increased decomposition and excretion prevail, decay commences. The shrivelled, almost mummy-dry forms of very old people, show how far the diminution of the body can go in advanced age.

Each period of life in the animal world shows a different degree of activity in the reception of elements and their union with each other. This law, which, in the different organs, uninterruptedly carries on the organism by nutrition and decay, and regulates the vital functions of the body, undoubtedly must control every single part, every fibre.

Although the views of several authors differ on the first perceptible beginnings of milk teeth, they agree in one point, viz. that in the third month of the human embryo-life the first commencements of these small organs can be recognised as very tender vesicles in the jaws, which themselves are still far from perfection. They exhibit the place, upon which the teeth will be formed hereafter, and, therefore, are equal to the cytoblastema, the origin of all texture. According to Goodsir, these vesicles originate from the folding in of the mucous membrane of the mouth; according to Rousseau, from the periosteum of the maxillary bones. The last of these two views has decidedly more physiological proofs of the truth, for it is more probable, that that portion of the gum which is close to the maxillary bone forms the vesicles, than Goodsir's idea, (that the vesicles originate from the exterior layer, the mucous membrane of the mouth.)

The tooth vesicle, which prolongates after the breaking through of the crowns and the beginning of the formation of the roots, and closely joins this last part of the tooth, appears as a periosteum of the root, and it is unaccountable, how during this period the mucous membrane should lose its own anatomico-physiological peculiarities, and change into periosteum. But, if I consider the teeth-capsules as formed out of the periosteum of the jaw-bone, and destined to accomplish the development of the teeth, organs which in chemical composition and excess of inorganic matter are most closely related to the bones, I believe with more reason than several others, who consider the teeth productions of the mucous membrane, that they are appendages of the maxillary bones, or rather of the periosteum thereof.

The periosteum supplies in all bones, through the extension of vessels to the bone cells, nutrition to the surface of the bone, and forms this layer during the period of development. Ought we not now, after this comparison, to believe that the formation of teeth is accomplished by the periosteum which forms bones instead of the mucous membrane, the more so as the physiological activity of the latter throughout the whole organism is only directed to the production from the many small glands, which are situated in this membrane, of the mucus for its own secreting surface, which mucus is destined to be an enveloping layer for the organs of respiration, digestion, generation, &c.? The teeth are products of a membrane, not however of a mucous membrane or an epidermis, as are nails and hair, but of the periosteum of the maxillary bones, which alone is able to generate a product so similar to bone.

According to the latest microscopical investigations, each tooth-germ appears to consist of three distinct tissues which are essentially different in their structure. The first outermost layer forms that sac, which, according to Rousseau, originates from the periosteum of the maxillary bone. This encloses the second and third tissues, which appear first about the end of the fourth month of embryo-life at the base of the tooth capsule, in form of the gelatinous tooth germ,* which is covered again at its surface by the enamel organ† in shape of a cap. At this time we already find six teeth-capsules in each jaw. The increasing activity of development of the embryo (consisting here in absorption and combination of elements) surpasses comprehension. So also in the development of the teeth. The tooth germ, originally packed away at the base of the tooth vesicle, presently enlarges through the absorption of new granules of this germ-like matter, and in the sixth month of embryo-life assumes the form of the crown of the future tooth, constituting still, covered by the enamel organ, the soft gelatinous frame-work of the tooth-crown.

As in the cartilages of the embryo, which become bones, the deposition of a much larger quantity of inorganic matter first takes place at the highest points, and advances from these, gradually hardening the soft tissues, so a similar process occurs in the first deposition of the genuine dentine; for after the completion of the before mentioned gelatinous frame-work of the crown, there originate as many small hard points as there are centres of formation of the dentine, and finally as many discs as the tooth has points. These apparently ossifying tops extend continually till they join each other, and after the dentine has first united the circumference of the crown in shape of a delicate thin envelop, they deposit from the exterior towards the interior new masses of that tubular structure which characterises the dentine. By this process the thickness of the crown increases to the cavity of the tooth wherein the vessels and nerves are imbedded.

Directly after the exterior layer of dentine is finished, the deposition of the enamel fibres commences upon the surface of

* Purkinje says, "In the tooth germ the granules are not connected by fibres of cellular tissue as is the case in the enamel pulp."

† The same author remarks : "The enamel organ, which is enveloped by the interior surface of the tooth sac, consists of a granular matter, which covers the pulp like a cap. The interior layer of the enamel organ, *membrana adamantina*, shows a quantity of closely compressed stripes, which represent the formative fibres of the enamel."

the crown, and the enamel grows from within outwards by the deposition of new fibres.

For a short time after their formation, the tooth capsules lie near each other without separating walls, and a later period only divides these germs by the commencement of the formation of the tooth-partitions. The inner and outer wall of the process forming the tooth partitions is formed from the maxillary bone, and the partitions will elongate, as the alveolar process approaches its completion. After birth, when the process of the tooth-partitions has reached a certain size, and the partitions allow sufficient space for the development of the roots, the deposition of dentine begins first upon the elongating pulps from without inwards, exactly as the crowns were formed.

Favored by advancing formation of the tooth-partitions the tooth-sacs close more and more upon the roots, become the periosteum of those parts of the tooth, and taking on the action peculiar to this membrane, begin to form that envelop of bone, which exists upon the roots after the formation of teeth is perfected. The enveloping layers of the teeth, the enamel on the crowns and the cement on the roots do not receive from the pulp any deposit of material, but are supplied with matter from the enamel organ and the periosteum of the root, which have to perform that duty. It has been asked, how the existence of these tissues can be explained on the supposition that there is no connection with the interior. There is no difficulty in the envelop of the roots, for the periosteum always embraces the root, and nutrition goes on uninterruptedly through the prolongations of vessels the periosteum sends towards the cement.

But how is it with the enamel, when the crown, after its eruption, still exists so long without any supply from without? Is it right to assume, that from that period a relation with the pulp takes place through the tooth bone, when according to all observations the tubular texture of the dentine is not perceptible beyond the limits of the enamel, to procure the imbibition for the enamel through those immensely fine tubes?

The elements of the enamel are according to Berzelius: phosphate of lime and fluoxyd of calcium 88.50; carbonate of lime

8; phosphate of magnesia 1.50 and scarcely 2 per cent. of animal matter. This unique peculiarity of composition in which almost the whole consists of inorganic elements makes it probable that this structure does not need the metamorphosis of matter for its existence. All the other elementary tissues of the body are more or less able to regenerate themselves completely or partially after a loss of substance, but I never found it mentioned or experienced myself, that enamel was able to repair its loss. Professor Hyrtl mentions in his work on human anatomy two cases of cured tooth fractures, the one a very instructive preparation in the Berlin Museum, and the other one in his own possession, the fracture of the neck of a front tooth, healed by callus. The particular remark, "healed by callus," points out, that the fracture must have happened within the limits of the bone envelop of the root. The inability of the enamel to reproduce itself furnishes a corroboration of this view.

Having established this point, that the enamel is thrown out of connection with the rest of the organism after the eruption of the crowns, it is a question, whether this hard, solid, envelop, not open for nutrition, limits the further growth of the crown.

The roots strengthen and elongate, but the circumference of the crowns remains the same.

I am prepared for the objection, that in many dyscrasies, the teeth show different colors, which change must be produced upon the enamel from the interior, and that, therefore, a connection must exist with the pulp. Dr. G. De Haven White mentions in this journal, (ix year, June,) in an article on anatomy and physiology of dentine, several cases occurring under his observation, in which teeth that lay for some time in a tincture of red saunders were found penetrated by this colored fluid.

I meet this objection by citing the pellucidity of the enamel, which allows the shining through of the discolored dentine.

The development of the remaining jaw teeth is accomplished by exactly the same process of capsule formation as in the case of the milk teeth. About the sixth month of embryo life we notice in the maxillaries, bones which in the mean time have proceeded in their development, the tooth sacs of the third jaw teeth, (first grinders.) Still later appear the germs of the fourth jaw teeth (second grinders) and about the fourth year of age those of the wisdom teeth.

The primitive development of the remaining first two jaw teeth differs from this. They are formed during the first year of life in the same order in which the milk teeth fall out. The delicate commencements of those substitute teeth are in relation with the milk teeth. The question, why there is a double set of germs has been answered in the clearest manner, as we now repeat. It was remarkable, that the perfect formation of all the milk teeth is finished in three years or little more, while that of the remaining teeth takes a period of 12 to 14 years, not even mentioning the late breaking through of the wisdom teeth. Until the seventh year of age the small jawbone of the child has not the size which is required by the remaining teeth for their reception, and which is marked by the size of their crowns.*

However there must exist until that time, when the proportional enlargement of the maxilla (through the general development of the organism) makes the reception of the remaining teeth possible, organs which shall masticate food, introduce nourishment and carry on the development of the organism. The wisdom teeth, the germs whereof are set so early, only break through about the time of perfect development of the whole organism, that is after the maxillary bones have reached their full size to allow the formation of said teeth.

The beginnings of the permanent teeth, after being formed in the appointed order, at first lie in the same tooth partitions with those of the milk teeth, and undergo a change of position at a somewhat later period; for, because the germs of the milk teeth are considerably in advance of the others in their development, the deposition of dentine upon the surface of the milk teeth has commenced for some time, and the formation of roots

*As it is known, the alveolar apophysis has room for the formation of only four incisors, two canines and the six first jaw teeth; so space is wanted for four more jaw teeth.

has proceeded after birth, the delicate germs of the remaining teeth press backwards to the posterior bony lamella of the maxilla, and here in small cavities develop the permanent teeth. A bony membrane, which originates from the base of the tooth partition now separates the hitherto common partitions of the milk and permanent teeth, so that only a small opening remains for communication, for both kinds of teeth have relation to each other through their vessels and nerves.

About the fifth year of age the development of the milk teeth reaches its highest point. The introduction of material diminishes, ceases, and the plastic elements of blood, which serve for new depositions and the preservation of the existing ones must be absorbed by the permanent teeth only, to produce their development. Some time before the falling out of the milk teeth the small bony membrane which arose from the base of the tooth partition disappears. Again, both kinds of teeth are in one division, and we notice, how the crowns of the permanent single rooted teeth press below and somewhat behind the milk teeth, but those of the teeth with several roots between the extensions of the roots without touching them. At a later period during the development of the roots of the permanent teeth and the closing up of their crowns the most tender pulling of the milk teeth vessels will be sufficient, to create in their almost capillary vessels, at least a partial interruption of circulation; for it cannot be proved, that the crowns of the permanent teeth exercise a purely mechanical power to push away the milk teeth; but even a partial interruption of circulation in the milk teeth vessels is sufficient to impede the support of the existing structure; for a decrease or change in the admission of blood to the members of the animal body produces a decrease of the existing quantity.

"Without change of matter, the life of the cell, which obtains nourishment from the surrounding germinal fluid, growth is impossible. Cells die, when they are separated from the mother soil, which contains the lymph, to which their fluid contents come into relation." (Moleschott.)

The appearances at the period of second dentition are twofold. Either nervous or inflammatory affections of very different degrees occur in the teeth, which ought to be removed, and last until those teeth fall out, or are removed artificially; or the milk teeth begin to go through, without pain, all the stages of decay to which each formation not accessible to nourishment is subjected. In the first case, hyperæmia of those organs takes place. The normal reaction does not keep time with the development of the permanent tooth, and the natural consequence is, that the milk tooth, supplied with heightened qualities of life by the increased affluence of blood, presents the reflection of each of these diseased appearances. But in the second case, the existence of the milk teeth ceases; the short life of those organs fails in the same proportion, in which that of the permanent teeth advances; and where the internal vital power dies, we notice changes in small and large objects, which show themselves by decrease of quantity.

The reaction begins with changed and diminished absorption of material and increased waste and loss. This proccss if it takes place at the same time in the different parts of the body, and prepares the living body for death, we call retrograde change of the living into dissolution.

The elementary principles by which everything in the body dissolves, when life closes through the altered proportion of ingestion and excretion of matter, must equally appear in the short life of the milk teeth, and as I remarked before, their decay and the perfect disappearance of their roots is not an extraordinary peculiar act, which does not take place anywhere else in the human organism. The eruption of teeth is scarcely more interesting.

About the sixth month of age, after the middle and lower front teeth have reached perfection, when the formation of roots goes on, which causes the steady growth of their crowns into the hollow cavity of the mouth, the single tooth partitions are still closed from within, outward, by the following formations, in uninterrupted order, above the alveolar apophysis: 1, the tooth vesicles; 2, the cartilaginous plate, which in sucking of the babe, serves to hold the nipple, and 3, the layer of cellular texture which unites this cartilage with the third layer of mucous membrane of the mouth.

All those parts of different density must be absorbed to permit the appearance of the milk teeth. Through the closing up of the teeth crowns towards the cavity of the mouth, first the tooth sacs and afterwards the enclosing layers, by the least pressure, suffer an interruption of circulation in their capillary vessels, and the blood—the mother fluid of all formations—cannot supply them with that matter, which is essential for the existence of those parts.

This process which attracts our attention not less, never has been mentioned as something similar by those who saw wonders in the absorption of the milk teeth.

Laymen might object, that the tooth sacs, the mucous membrane and even the cartilage plate are of immensely less density, than the roots of bony matter and dentine. I, however, answer, that aneurisms which enclose a bone are able to cause absorption, where this enlarged arterial vessel comes in contact with the hard matter. Does the absorption of milk teeth roots, by its waving fluctuation up and down, stand isolated now, when an artery can effect a decrease of bony matter? And where in the loss of bony matter shall we find that particular organ, which undertakes the removing of the milk tooth roots, and according to some, is only formed and active at this period? In science the absorption is explained in the following manner:

The textures of every kind in the animal organism have direct or indirect relation to the blood,* for it forms every thing. If now by pressure or changed activity of nerves an interruption of supply takes place in the cells of a tissue, the imbibition and exhalation, which are necessary to nourish the most delicate fibres, are at an end in that part. The cell dies and still is amongst the neighboring living particles. Where, however, every thing lives, this dead particle cannot remain. According to the same law, by which they can absorb matter from the blood the adjoining cells absorb this decaying part, for the dying vital power can exercise no reaction. So each particle in which,

*Perhaps with the single exception of the enamel. *Author.*

for causes already assigned, the ingestion of matter ceases, will be absorbed as a dead body by the living ones, and one cell takes from another one the substances which constituted its former life, to die itself and be carried off from the field of life, when the destruction reaches it.

Notwithstanding several positive dogmas it is based upon scientific logic, that the crowns of the permanent teeth touch the roots of the milk teeth, where this part of the tooth is found absorbed; and all the authors who hold the opinion that the expulsion of the milk teeth takes place, not by purely mechanical power, surely only mean hereby the single active power of pressure. It may be that the cause of tooth eruption generally is the roots; it may be that the active power of eruption is caused by the enlargement of the maxillary bones, the alveolar apophysis which are developed from the main body, and by such means cause the teeth lying in the tooth partitions to rise and favor their eruption; or finally it may be, that both those causes unite for this purpose, it appears sure to me, that the roots undergo through the enumerated causes a loosening of connection with the maxilla besides the interrupted nutrition of dentine of the milk teeth before their falling out. This, however, must cause an interruption in the life of the delicate vessel extensions, which lead from the enclosing periosteum of the tooth-partitions to that and the bone cells of the roots, and here also the want of supply, which in the bony enclosure of the roots takes place from out to inside, causes a decrease of quantity.

In the living organism, where the many different formations originate out of a fluid—the blood—the powers of attraction and repulsion, of ingestion and excretion are based upon reciprocity. Nowhere appears a single operating power. If, therefore, I suppose the absorption of the milk tooth roots to be caused by the above mentioned powers, I think I remain true to the elementary principles, which constitute the life of beings generally. No process in the organism stands isolated; one is based upon another, and each one finds its explanation not specially, but generally.

I leave it to the judgment of experienced and competent men, how far these observations can compare with the known dogmas of science. Kindly shall I receive any proof of error based upon physiological principles.

